# A universal gene correction approach for FKRP-associated dystroglycanopathies to enable autologous cell therapy

**DOI:** 10.1016/j.celrep.2021.109360

**Published:** 2021-07-13

**Authors:** Neha R. Dhoke, Hyunkee Kim, Sridhar Selvaraj, Karim Azzag, Haowen Zhou, Nelio A.J. Oliveira, Sudheer Tungtur, Carolina Ortiz-Cordero, James Kiley, Qi Long Lu, Anne G. Bang, Rita C.R. Perlingeiro

**Affiliations:** 1 Lillehei Heart Institute, Department of Medicine, University of Minnesota, Minneapolis, MN, USA; 2 Stem Cell Institute, University of Minnesota, Minneapolis, MN, USA; 3 Conrad Prebys Center for Chemical Genomics, Sanford Burnham Prebys Medical Discovery Institute, La Jolla, CA, USA; 4 McColl-Lockwood Laboratory for Muscular Dystrophy Research, Cannon Research Center, Carolinas Medical Center, Atrium Health, Charlotte, NC, USA; 5 Lead contact

## Abstract

Mutations in the fukutin-related protein (*FKRP*) gene result in a broad spectrum of muscular dystrophy (MD) phenotypes, including the severe Walker-Warburg syndrome (WWS). Here, we develop a gene-editing approach that replaces the entire mutant open reading frame with the wild-type sequence to universally correct all FKRP mutations. We apply this approach to correct FKRP mutations in induced pluripotent stem (iPS) cells derived from patients displaying broad clinical severity. Our findings show rescue of functional α-dystroglycan (α-DG) glycosylation in gene-edited WWS iPS cell-derived myotubes. Transplantation of gene-corrected myogenic progenitors in the FKRP^P448L^-NSG mouse model gives rise to myofiber and satellite cell engraftment and, importantly, restoration of α-DG functional glycosylation *in vivo*. These findings suggest the potential feasibility of using CRISPR-Cas9 technology in combination with patient-specific iPS cells for the future development of autologous cell transplantation for FKRP-associated MDs.

## In brief

Dhoke et al. develop a universal CRISPR-Cas9 gene-editing strategy for the correction of FKRP mutations. Myogenic progenitors from gene-edited WWS patient-specific iPS cells show rescue of phenotype *in vitro* and upon transplantation into muscles of FKRP mutant mice.

## Graphical Abstract

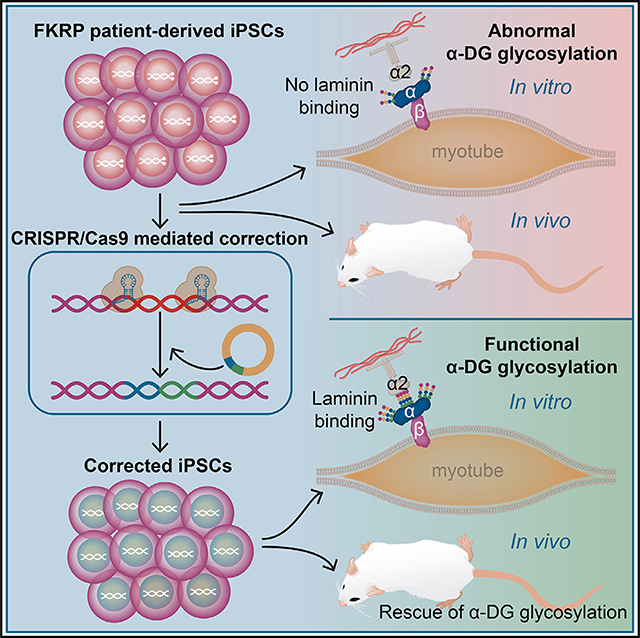

## INTRODUCTION

Mutations in fukutin-related protein (FKRP) are associated with muscular dystrophies (MDs) of remarkably variable clinical severity, ranging from mild limb-girdle MD type 2I (LGMD2I, also known as LGMDR9) to severe forms of congenital MD (CMD), including Walker-Warburg syndrome (WWS) and muscle-eye-brain disease (MEB) (Beltran-Valero de Bernabé et al., 2004; Mercuri et al., 2003; Topaloglu et al., 2003). Despite the vast clinical spectrum, all FKRP-associated diseases share a common biochemical defect, hypoglycosylation of α-dystroglycan (α-DG), and therefore are commonly referred to as dystroglycanopathies (Mercuri et al., 2003; Michele et al., 2002; Muntoni et al., 2002). Besides FKRP, mutations in several other genes directly involved in α-DG glycosylation, such as POMT1, POMT2, LARGE, and FKTN, result in different forms of dystroglycanopathies (Beltrán-Valero de Bernabé et al., 2002; Martin, 2007). α-DG is a component of the dystrophin-glycoprotein complex that links the actin cytoskeleton of the muscle fibers with extracellular matrix (ECM) ligands, including laminin α2, perlecan, neurexin, and agrin (Gee et al., 1994; Gomez Toledo et al., 2012; Yoshida-Moriguchi and Campbell, 2015; Yoshida-Moriguchi et al., 2010), and is therefore critical for maintaining the integrity of the muscle fiber (Ervasti and Campbell, 1993). As a result of FKRP mutations, α-DG glycosylation is impaired, resulting in the disruption of the interaction between the cytoskeleton and the ECM, which leads to the loss of cell membrane integrity and, ultimately, fiber damage and progressive muscle degeneration (Awano et al., 2015; Brockington et al., 2001; Louhichi et al., 2004; Topaloglu et al., 2003).

There is no effective treatment for FKRP-associated dystroglycanopathies. To date, most studies have focused on adeno-associated virus (AAV)-mediated gene therapy. Although encouraging results have been reported, these studies are still in the early stages (Gicquel et al., 2017). Cell-based therapies represent another potential option for treating FKRP-associated MDs. Pluripotent stem (PS) cells are particularly attractive because of their unique expansion and differentiation potential as well as their amenability to genetic modification. Several studies have documented the *in vivo* regenerative potential of PS cell-derived skeletal myogenic progenitors (Darabi et al., 2012; Goudenege et al., 2012; Tedesco et al., 2012), and therefore, the combination of patient-specific induced PS (iPS) cells with gene-editing approaches provides an opportunity for autologous cell transplantation (Selvaraj et al., 2019b). The gene correction of α-sarcoglycan (Tedesco et al., 2012), dystrophin (Young et al., 2016), and calpain 3 (Selvaraj et al., 2019a) mutant patient-specific iPS cells led to the rescue of phenotype, suggesting the potential for autologous cell-based therapy in these MDs. Despite these successes, a major hurdle with genetic-correction approaches is that the protein coding sequence is typically distributed over many exons, spanning large distances; thus, specific mutations require tailored approaches. The *FKRP* gene is unusual in that the entire open reading frame is contained within a single exon (exon 4). Taking advantage of this providential genetic architecture, we developed an exon 4 replacement approach that is suitable for the correction of any point mutation in *FKRP*. We show the rescue of functional α-DG glycosylation *in vitro* and *in vivo*, thus demonstrating the feasibility of this universal approach to rescue the muscle phenotype associated with FKRP mutations.

## RESULTS

### *In vitro* phenotype of FKRP mutant patient-specific iPS cell-derived myotubes

FKRP mutant iPS cell lines were generated from three patients displaying broad clinical severity. As outlined in Figure S1A, FP4 iPS cells were generated from a WWS patient, CDI73 iPS cells were derived from a LGMD2R9 patient, and FP3 iPS cells were generated from a patient with an intermediate phenotype LGMDR9/CMD. Genetic analysis revealed heterozygous FKRP mutations for the FP4 (c.558dupC; c.1418T>G) and FP3 (c.217C>T; c.826C>A) samples and homozygous mutation for the CDI73 patient (c.826C>A) (data not shown). Characterization of these iPS cell lines confirmed normal iPS cell morphology, expression of pluripotency markers, normal karyotype, and development of teratomas containing cell types from all three germ layers upon their injection into immunodeficient mice (Figure S1B) (Ortiz-Cordero et al., 2021b).

**Figure 1.**
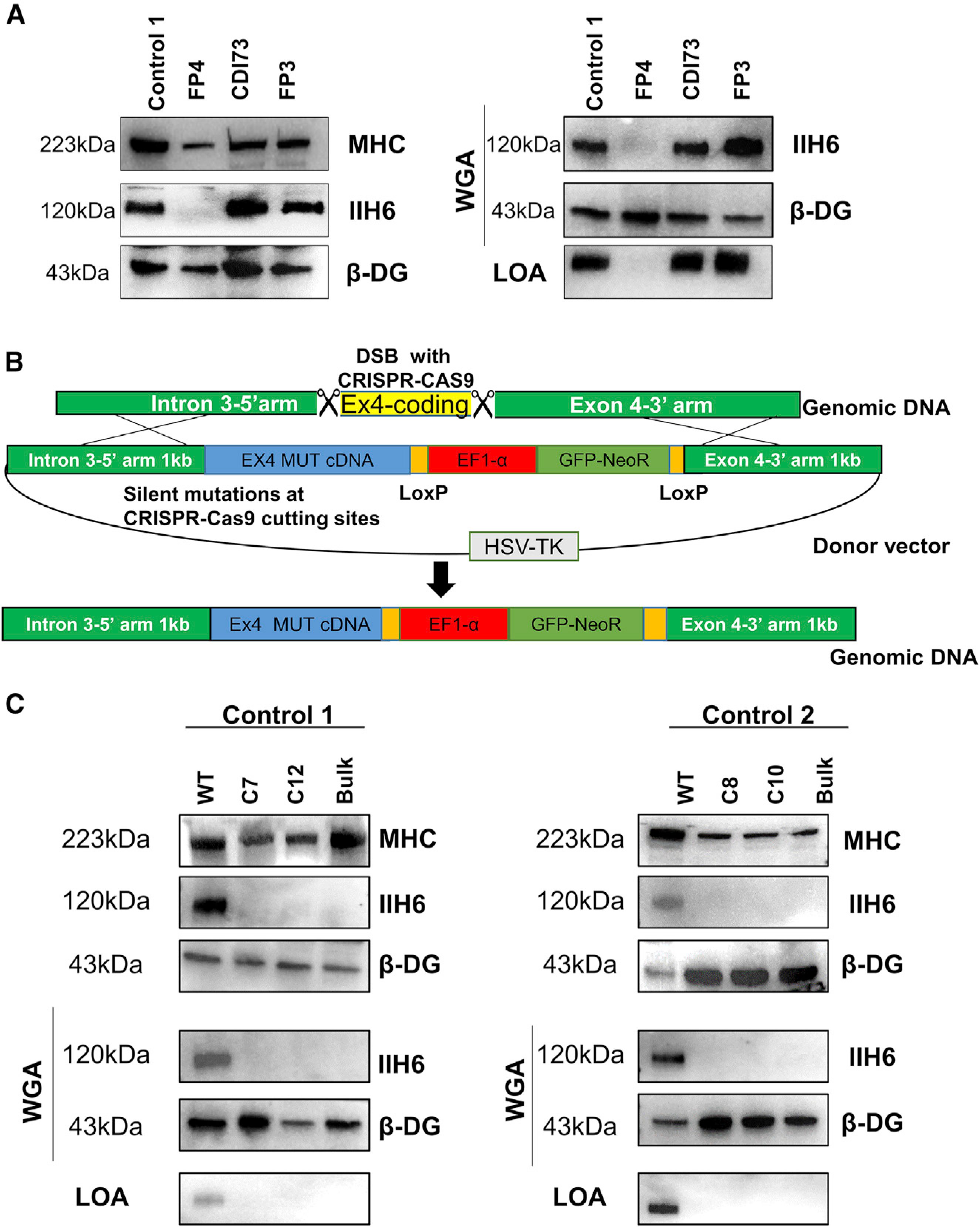
Phenotype of FKRP mutant myotubes derived from patient-specific and isogenic iPS cells (A) Representative western blot for IIH6 in myotubes from FKRP mutant patient-specific and control iPS cell lines. MHC and β-DG were used as differentiation and loading controls, respectively. The right panel shows wheat germ agglutinin (WGA) pull-down for these samples and representative laminin overlay assay (LOA) of WGA elutes. (B) Scheme shows the gene-editing strategy used to insert a WWS-associated FKRP mutation in control iPS cell lines. (C) Western blot for IIH6 in myotubes from WWS FKRP mutant isogenic iPS cell lines. Results from control 1 (WT, mutant bulk, and clones C7 and C12) and control 2 (WT, mutant bulk, and clones C8 and C10) iPS cell lines are shown in the left and right panels, respectively. MHC and β-DG were used as differentiation and loading controls, respectively. The center panel shows the IIH6 immunoblot in WGA pull-downs. Representative LOA of WGA elutes shows loss of laminin binding in WWS-associated FKRP mutant myotubes (lower panels).

Since the three iPS cell lines are associated with varying disease severity, we next sought to evaluate the levels of functional α-DG glycosylation in these patient samples using the IIH6 monoclonal antibody, which specifically recognizes the laminin-binding domain of α-DG. Using the conditional expression of PAX7, we differentiated FKRP mutant and unaffected (control 1) iPS cells into myogenic progenitors, and subsequently into myosin heavy-chain (MHC)-expressing myotubes. As shown by western blot (Figure 1A) and immunofluorescence staining (Figure S1C) assays, FP4 myotubes (severe WWS) are devoid of IIH6 immunoreactivity, whereas CDI73 and FP3 myotubes show IIH6 positivity similar to control counterparts. Importantly, the laminin overlay assay (LOA) revealed the inability of FP4 myotubes to bind laminin (Figure 1A, left panel), further confirming the lack of α-DG functional glycosylation in WWS cells. Similar results were observed at the iPS cell stage, as assessment of IIH6 immunoreactivity by flow cytometry and western blot as well as LOA showed impaired α-DG functional glycosylation only for FP4 iPS cells (Figures S1D and S1E). Therefore, our results show that only the severe WWS sample (FP4) displays a clear defective phenotype *in vitro*. This is in agreement with previous studies performed in primary samples from patients with FKRP mutations, which documented variable α-DG hypoglycosylation in CMD and LGMDR9, in contrast to WWS, which consistently showed the complete absence of functional α-DG glycosylation (Kava et al., 2013; Louhichi et al., 2004; Muntoni et al., 2007).

### Generation of FKRP mutant WWS isogenic iPS cells

To further confirm that the lack of α-DG functional glycosylation in the WWS patient sample is specifically due to the FKRP mutations, we next generated WWS isogenic iPS cell lines by inserting the well-established FKRP mutation c.953G>A, (p.318C>Y) (Beltran-Valero de Bernabé et al., 2004) into control unaffected iPS cells. Since the entire open reading frame of FKRP is within the exon 4 of the gene, we targeted this exon for the gene knockin. Using CRISPR-Cas9, we first created two double-stranded breaks (DSBs) in exon 4 to remove the entire coding region, followed by homology directed repair (HDR) from an exogenous donor vector to allow gene knockin of the mutant coding region carrying the c.953G>A FKRP mutation, along with SV40 poly A signal sequence and a loxP-flanked selection cassette (Figure 1B). Following the transfection of CRISPR-Cas9 ribonucleoprotein (RNP) complexes and HDR donor plasmids, iPS cells were cultured with G418 to select for neomycin-resistant cells, which are positive for the knockin. To select against non-specific integration, we treated cells with ganciclovir to eliminate cells expressing the thymidine kinase gene from a constitutive promoter in the donor vector backbone. Since functional α-DG glycosylation can be detected at the iPS cell stage, following antibiotic selection, mutant iPS cells were further purified by fluorescence-activated cell sorting (FACS) based on negativity for IIH6 staining (Figure S2A). IIH6^neg^ cells were expanded and subjected to single-cell cloning. Two clones were isolated for each of the two control isogenic iPS cell lines (C7 and C12 for control 1, and C8 and C10 for control 2), and these were found homogeneously negative for IIH6 immunoreactivity (Figure S2A). Sequencing analysis confirmed the homozygous knockin of the FKRP c.953G>A mutation in all isogenic iPS cell clones (Figure S2B). Next, we differentiated FKRP mutant and control isogenic iPS cell lines into myotubes and assessed the *in vitro* phenotype. Lack of IIH6 immunoreactivity confirmed the loss of α-DG functional glycosylation in FKRP mutant isogenic iPS cell lines (Figures 1C and S2C). This was further established by their inability to bind laminin (Figure 1C, lower panels), thus confirming that WWS-associated FKRP mutations result in the complete loss of α-DG functional glycosylation.

### *In vivo* phenotype of WWS FKRP mutants

**Figure 2.**
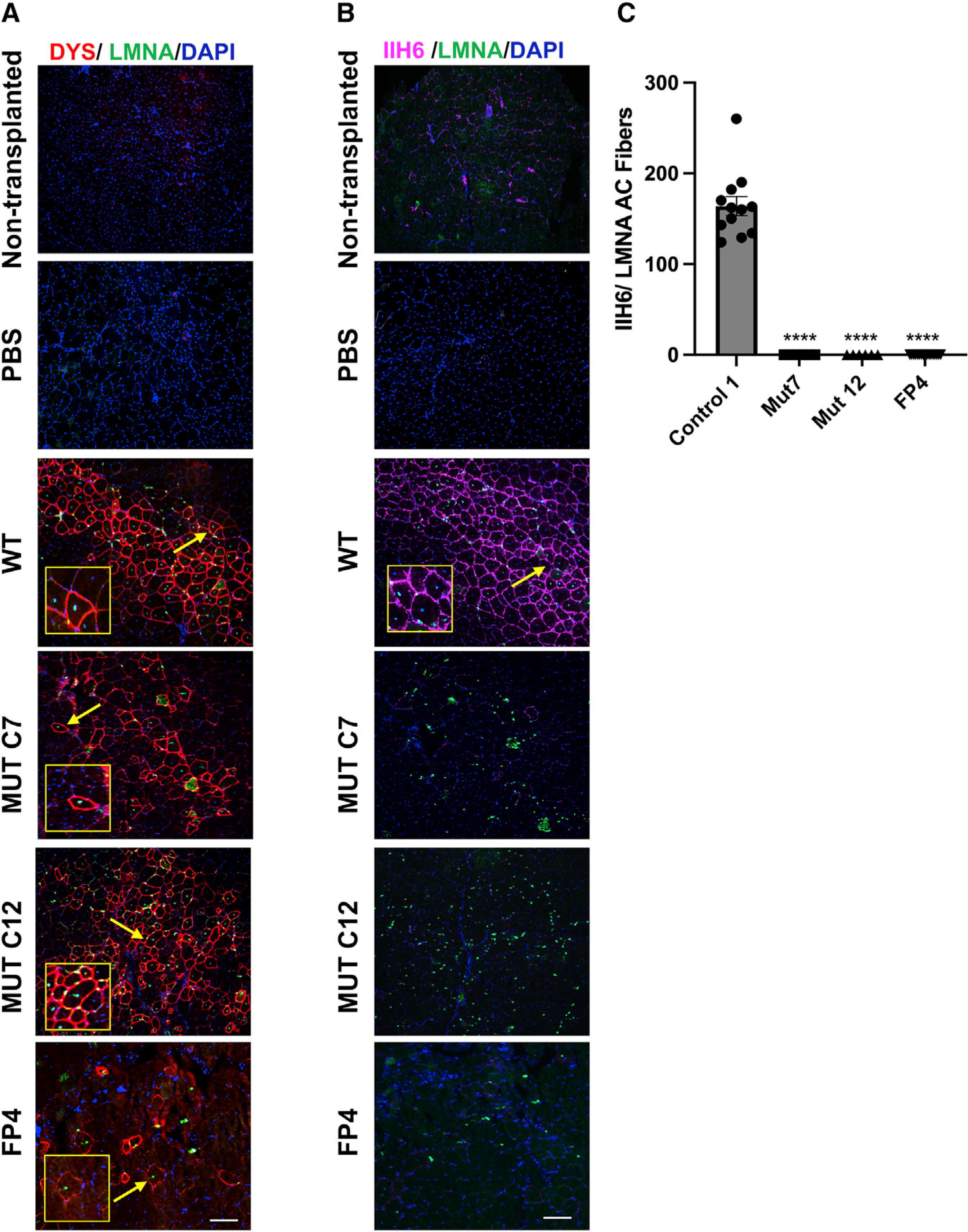
Validation of WWS phenotype *in vivo* Representative images show the engraftment of myogenic progenitors derived from WWS FKRP mutant isogenic (WT and mutant clones C7 and C12) and FP4 patient-specific iPS cell lines upon their transplantation into TA muscles of FKRP^P448L^-NSG mice. (A) Immunostaining for human DYSTROPHIN (DYS, in red) and human LAMIN A/C (LMNA, in green). DAPI in blue stains nuclei. PBS and non-transplanted muscle served as negative controls. Scale bar, 200 μm. (B) Immunostaining for IIH6 (in purple) in combination with LMNA (in green). DAPI stains nuclei (in blue). PBS and non-transplanted muscle served as negative controls. Scale bar, 200 μm. (C) Graph shows the quantification of the total number of donor-derived IIH6^+^/LMNA^+^ myofibers in TA muscles that had been transplanted with myogenic progenitors differentiated from control 1 (n = 12), MUT C7 (n = 4), MUT C12 (n = 4), and FP4 (n = 18) iPS cells. Data are shown as means ± SEMs. ****p < 0.0001 by ANOVA followed by the Tukey’s test.

To determine whether lack of functional α-DG glycosylation can be recapitulated *in vivo*, we transplanted myogenic progenitors derived from the FP4 patient-specific mutant as well as from the isogenic FKRP mutant (c.953G>A) iPS cell lines into cardiotoxin pre-injured tibialis anterior (TA) muscles of FKRP^P448L^-NSG mice (Azzag et al., 2020). These mice were generated by crossing the established FKRP^P448L^ model (Chan et al., 2010) to immunodeficient NSG mice with the intention of creating an ideal model for the transplantation of human cells in the context of FKRP mutation. FKRP^P448L^-NSG mice are characterized by the absence of α-DG functional glycosylation, similar to their immunocompetent FKRP^P448L^ counterparts (Chan et al., 2010), and lack of B, T, and natural killer (NK) cells (Azzag et al., 2020). Pre-injury is commonly used before cell transplantation when using both wild-type (WT) and dystrophic mice to enhance the regenerative response, thus providing a better assessment of the repopulation potential of injected cells (Arpke et al., 2013; Bentzinger et al., 2014; Darabi et al., 2008; Judson et al., 2018), which is even more relevant in the context of xenotransplantation (Azzag et al., 2020). Six weeks post-transplantation, using human-specific antibodies for LAMIN A/C (LMNA) and DYSTROPHIN, we observed donor-derived myofibers in mice that had been transplanted with myogenic progenitors from WT iPS cells and their respective isogenic FKRP mutant counterparts, as well as from FP4 patient-specific iPS cells (Figure 2A), but only the WT cohort showed significant positivity for IIH6 (Figures 2B and 2C). Transplantation of FKRP mutant myogenic progenitors resulted in LMNA^+^ myofibers devoid of IIH6 immunoreactivity (Figures 2B and 2C). As expected, PBS-injected and non-transplanted muscles showed no signal for human markers (Figures 2A and 2B), and only limited IIH6 background immunoreactivity was observed (Figure 2B), as previously described (Azzag et al., 2020). These results confirm that myofibers derived from the transplantation of WWS FKRP mutant myogenic progenitors lack functional α-DG glycosylation, validating this model for future rescue studies.

### Gene correction rescues functional α-DG glycosylation *in vitro*

**Figure 3.**
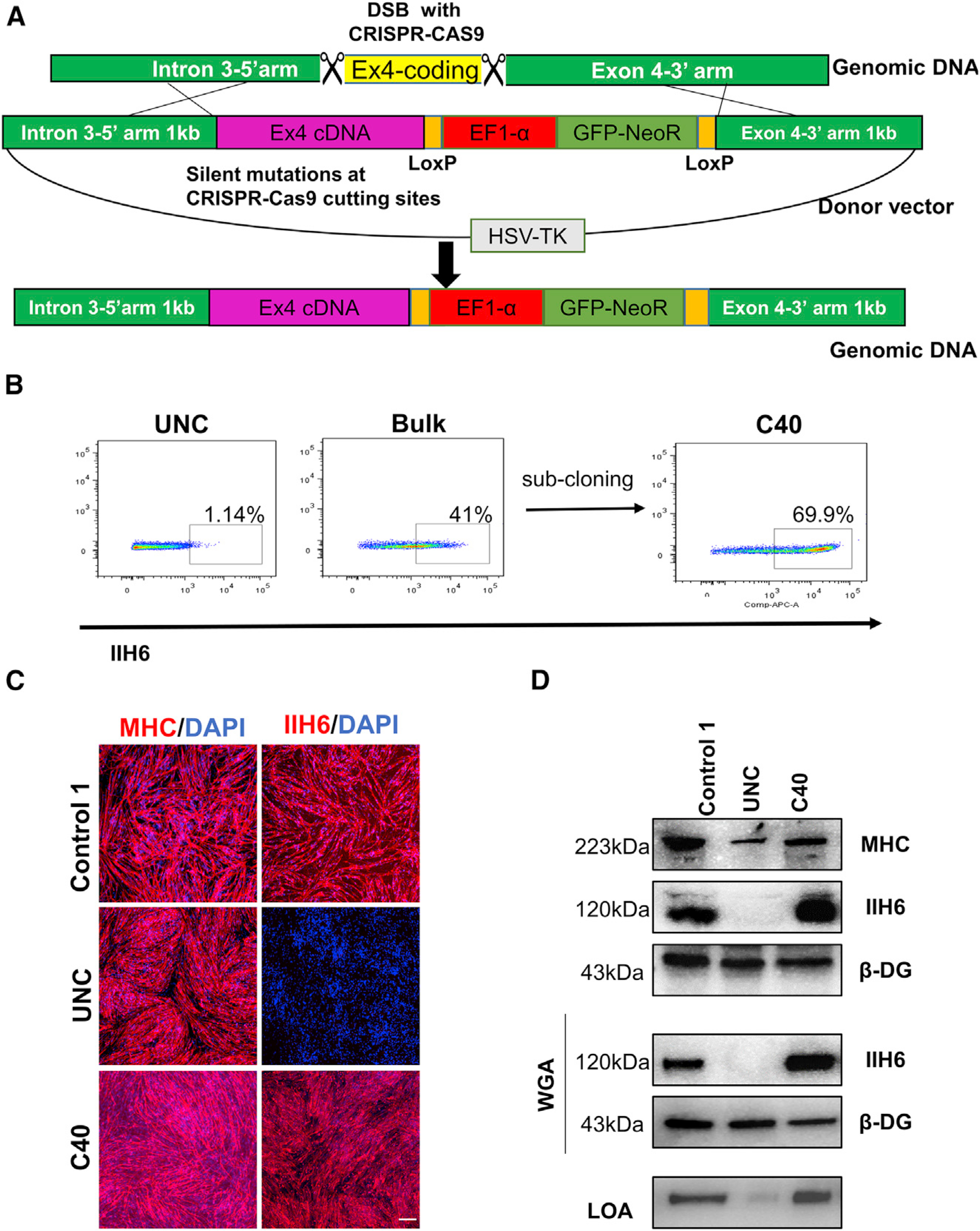
Gene correction of FP4 patient-specific iPS cells (A) Schematic of the universal gene-editing strategy for correction of FKRP mutations through HDR-based gene knockin. (B) Flow cytometry analysis for IIH6 in uncorrected and corrected (bulk and C40) FP4 iPS cells. (C) Representative images show immunostaining for MHC (left) and IIH6 (right) in myotubes derived from gene-corrected FP4 (C40) iPS cells. Myotubes from uncorrected FP4 and control 1 iPS cells served as negative and positive controls, respectively. DAPI stains nuclei (in blue). Scale bar, 200 μm. (D) Western blot and LOA show rescue of functional α-DG glycosylation in gene-corrected FP4 (C40) iPS cell-derived myotubes, as shown by IIH6 positivity and laminin binding (lower panel). MHC and β-DG were used as differentiation and loading controls, respectively.

Having established an *in vitro* and *in vivo* phenotype for WWS-associated FKRP mutations, next we developed a universal gene-correction approach for all FKRP mutations. As shown in Figure 3A, we created two DSBs using CRISPR-Cas9 to delete the entire mutant FKRP coding region in exon 4, followed by HDR from an exogenous vector to knock in the FKRP WT coding sequence. Upon lipofection with CRISPR-Cas9 RNP complexes and donor vector, FP4 iPS cells were cultured with G418 and ganciclovir to select for cells positive for the insertion and against non-specific integration, respectively. Since our earlier characterization showed that α-DG functional glycosylation can be detected at the pluripotent stage, we used FACS to further purify gene-edited iPS cells. Cells that survived the double antibiotic selection were sorted based on IIH6 positivity and initially cultured as a bulk population (Figure 3B). PCR amplification of the region spanning the knockin revealed that the knockin was homozygous (Figure S3A), and as expected, this was also the case for the 30 clones we analyzed (data not shown). We chose clone 40, which showed >70% positivity for IIH6 (Figure 3B) for further studies. Genomic DNA PCR (Figure S3A) and gene expression analysis for FKRP mRNA (Figure S3B) showed that only gene-corrected iPS cells displayed amplification for FKRP using knockin-specific primers. Sequencing further verified the gene correction of FP4 iPS cells (Figure S3C). Most important, we observed the rescue of functional α-DG glycosylation in gene-edited FP4 iPS cell-derived myotubes, as shown by immunofluorescence staining and immunoblotting for IIH6, and their ability to bind laminin (Figures 3C and 3D).

Next, we applied this universal gene-editing approach to CMD (FP3) and LGMD2I (CDI73) iPS cells, which resulted in clones positive for the gene knockin, as indicated by genomic DNA PCR amplification of the knockin-specific region (Figure S3D) and the expression of *FKRP* mRNA specifically from the gene knockin sequence (Figure S3E). Rescue of phenotype was not possible since FP3 and CDI73 samples do not display impaired α-DG functional glycosylation *in vitro*, but sequencing analysis validated the correction of gene-edited FP3 and CDI73 iPS cells (Figures S3F and S3G), confirming that this strategy can be used to correct any FKRP mutation.

Since the presence of GFP and neomycin resistance transgenes, or of any scar, such as the loxP sequence, are not desirable for future therapeutic applications, we constructed a donor vector devoid of the selection cassette, as well as SV40 polyA and loxP sequences (Figure S4A). A scar-free gene-editing approach is advantageous as it does not leave a selection marker or silent mutations in the genomic DNA (Ikeda et al., 2018). This scar-free donor vector was transfected along with CRISPR-Cas9 RNP in FP4 iPS cells, and resulting gene-edited FP4 iPS cells were purified by FACS based on IIH6 positivity (Figure S4B). The expansion of IIH6^+^ sorted cells resulted in enrichment for IIH6 (Figure S4B), and sequencing analysis further corroborated gene correction (Figure S4C). We confirmed the rescue of functional α-DG glycosylation in FACS-purified gene-edited FP4 iPS cell-derived myotubes by IIH6 immunoblotting and laminin-binding activity (Figure S4D). These data demonstrate the amenability of adapting our universal gene correction to a selection cassette- and scar-free approach, which is relevant for therapeutic application.

### Off-target activity

To determine the safety of gene-edited iPS cells, we assessed karyotypic stability and potential off-target mutations. Whole chromosomal analysis showed that genome editing did not affect the karyotype of gene-corrected patient-specific FKRP iPS cells (Figure S4E). We used the Off-Spotter software (Pliatsika and Rigoutsos, 2015) to identify the top 5 predicted off-target sites for each of the two guide RNAs used for FKRP gene editing. The genomic regions of these predicted off-targets were amplified by PCR in the corrected iPS cells and the uncorrected counterparts for all three patient-specific iPS cell lines. Using the ICE algorithm (Hsiau et al., 2019), sequencing chromatograms were analyzed to determine the percentage of off-target mutations. These analyses showed no detectable off-target activity (Table S1). Thus, these results suggest that the universal CRISPR-Cas9 gene-correction approach developed for FKRP mutant iPS cells is potentially safe, but a more comprehensive assessment will be required for future therapeutic applications.

### *In vivo* rescue of α-DG functional glycosylation upon transplantation of gene-corrected myogenic progenitors

**Figure 4.**
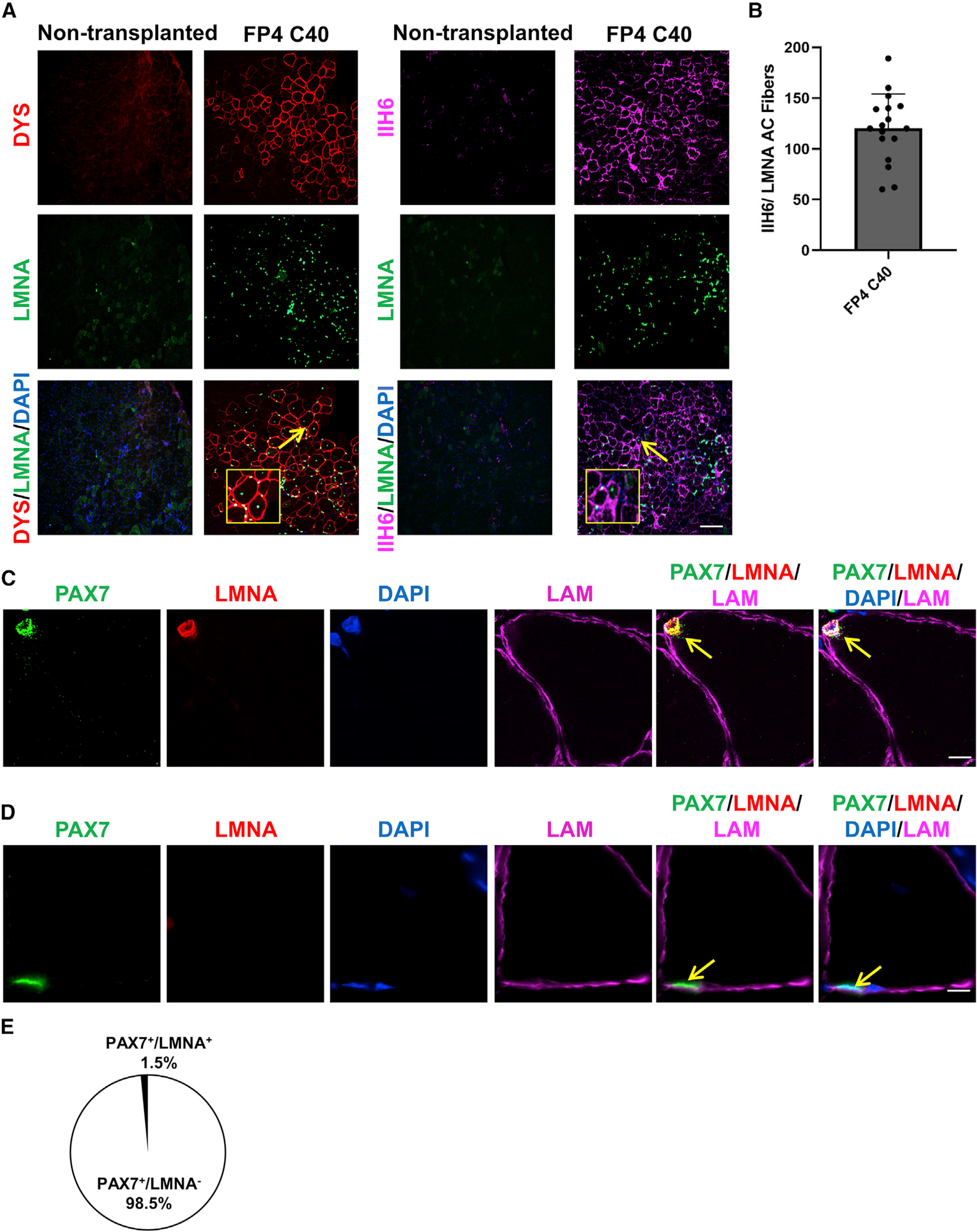
Transplantation of gene-corrected FP4 iPS cell-derived myogenic progenitors into FKRP mutant mice rescues functional glycosylation of α-DG (A) Representative images show the engraftment of gene-corrected FP4 (C40) iPS cell-derived myogenic progenitors following their transplantation into TA muscles of FKRP^P448L^-NSG mice. The left panel shows immunostaining for human DYS (in red) and human LMNA (in green), whereas the right panel shows immunostaining for IIH6 (in purple) in combination with LMNA (in green). PBS and non-transplanted muscle served as negative controls. DAPI stains nuclei (in blue). Scale bar, 200 μm. (B) Graph shows the total number of donor-derived myofibers, IIH6^+^/LMNA^+^, in TA muscles that had been transplanted with myogenic progenitors differentiated from gene-corrected FP4 (C40) iPS cells. Data are shown as the mean of 3 independent transplantation experiments ± SEM (n = 18 mice). (C and D) Immunofluorescence staining for satellite cells. (C) Representative images show donor-derived satellite cell engraftment in TA muscles transplanted with gene-edited FP4 (C40) iPS cell-derived myogenic progenitors, as shown by the presence of human LMNA^+^ (in red)/PAX7^+^ (in green) cells localized beneath the basal lamina (upper panel). (D) Representative images indicate a recipient PAX7^+^/LMNA^−^ satellite cell. Scale bar, 10 μm. (E) Percentage of PAX7^+^/LMNA^+^ (donor-derived) and PAX7^+^/LMNA^−^ (recipient-derived) cells per muscle section in mice that had been transplanted with gene-edited FP4 (C40) iPS cell-derived myogenic progenitors. Data are shown as means ± SEMs (n = 8 mice).

To determine the rescue of functional α-DG glycosylation *in vivo*, we transplanted gene-corrected FP4 iPS cell-derived myogenic progenitors in FKRP^P448L^-NSG mice. Our results show the presence of human donor-derived myofibers double positive for LMNA and IIH6, thus denoting the rescue of α-DG functional glycosylation in engrafted muscles (Figures 4A and 4B). It remains to be determined whether this level of α-DG functional glycosylation rescue is accompanied by functional improvement.

Next, we determined whether gene-edited patient-specific myogenic progenitors were endowed with the ability to seed the muscle stem cell compartment, as previously reported with myogenic progenitors derived from unaffected human PS cells (Darabi et al., 2012; He et al., 2020; Magli et al., 2017). As shown in Figure 4C, we observed the presence of human donor-derived LMNA^+^ cells occupying the satellite cell position and co-expressing the muscle stem cell marker Pax7 (Seale et al., 2000). Quantification of LMNA^+^Pax7^+^ cells revealed a contribution of ~1.5% of human donor-derived cells to the satellite cell pool (Figure 4E). As anticipated, the vast majority of the Pax7^+^ cell fraction was from the host and therefore negative for LMNA (Figures 4D and 4E). This level of engraftment is consistent with human-to-mouse transplantation (Kim et al., 2021), and the use of non-irradiated muscles, in which the recipient’s satellite cell compartment is preserved.

Our results demonstrate that transplanted gene-corrected FP4 iPS cell-derived myogenic progenitors give rise to α-DG functionally glycosylated myofibers and donor-derived satellite cells.

## DISCUSSION

The ability to derive myogenic progenitors with *in vivo* regenerative potential from human iPS cells (Darabi et al., 2012; Goudenege et al., 2012; Hicks et al., 2018; Rao et al., 2018) combined with the significant recent advances in genome editing technologies (Cong et al., 2013; Mali et al., 2013), enables the potential development of autologous cell therapy for MDs (Selvaraj et al., 2019a, 2019b; Tedesco et al., 2012; Young et al., 2016). A requirement for the development of iPS cell-based autologous cell therapy for MDs such as FKRP-associated dystroglycanopathies is the correction of underlying genetic mutations before the generation of myogenic progenitors. Here, we report the development of a universal CRISPR-Cas9-based gene-correction approach that could correct virtually all FKRP mutations. Our strategy involves complete excision of the mutant FKRP coding region in exon 4, replacing it with the WT FKRP coding region through HDR-mediated gene correction. We demonstrate that this approach corrects FKRP mutations in three different patient-specific iPS cell lines. Importantly, when applied to severely affected WWS iPS cells, this universal FKRP gene-correction approach resulted in the restoration of functional α-DG glycosylation in *in vitro*-generated gene-corrected iPS cell-derived myotubes and *in vivo* upon the transplantation of gene-corrected myogenic progenitors into FKRP^P448L^-NSG mice. Also of relevance, transplanted gene-corrected myogenic progenitors seeded the satellite cell pool, and due to the lack of ablation of host satellite cells and the xenotransplantation nature of engraftment, the percentage of human Pax7^+^ cells occupying the satellite cell niche is relatively low.

As mentioned in the Results, although the classic hallmark of FKRP-associated dystroglycanopathies is reduction in functional α-DG glycosylation, several studies have documented that patients with LGMD2R9 displaying the most common mutation c.826C>A (Alhamidi et al., 2017; Lee et al., 2019) and patients with CMD (Louhichi et al., 2004) show variability in the levels of glycosylated α-DG. This is in accordance with our results, as we did not observe a significant difference in the levels of functional α-DG glycosylation in LGMD2R9 and LGMD2R9/CMD iPS cell-derived myotubes compared to WT controls. Of note, we have observed similar results in iPS cell-derived myotubes and respective fibroblast samples from another cohort of three LGMD2R9 patients carrying the same mutation (data not shown). Previous studies have shown that autophagy and endoplasmic reticulum (ER) stress are elevated in LGMD2R9 muscle (Boito et al., 2007; Franekova et al., 2021; Lin et al., 2011), suggesting that additional mechanisms, other than the impairment of α-DG glycosylation and the disruption of its interaction with laminin, may contribute to the pathogenesis of FKRP-associated dystroglycanopathies. In any case, these warrant further investigation.

Although significant progress has been made in understanding the function of FKRP in the context of MDs (Gerin et al., 2016; Henriques et al., 2019; Kuwabara et al., 2020; Ortiz-Cordero et al., 2021a), limited treatment options are available for this group of MDs. AAV-mediated overexpression of the glycosyltransferase LARGE has been tested, but this led to the hyperglycosylation of α-DG, and may be the cause of the detrimental effects observed in treated muscles (Brockington et al., 2010; Vannoy et al., 2014; Whitmore et al., 2014). The use of AAV9 has allowed for the systemic delivery of fully functional FKRP to muscle cells of FKRP mutant mouse models (Qiao et al., 2014; Tucker et al., 2018; Xu et al., 2013). Despite progress and excitement, AAV-mediated gene therapy still faces several challenges, in particular, regarding the immune response to AAV capsids and/or transgenes (Colella et al., 2017; Li and Samulski, 2020; Mingozzi and High, 2013). Moreover, specifically for FKRP, recent studies have suggested that the levels of FKRP expression may need to be controlled to avoid toxicity (Gicquel et al., 2017). Given the recent identification of FKRP as a ribitol-5-phosphate transferase (Kanagawa et al., 2016; Kuwabara et al., 2020), the therapeutic effect of ribitol supplementation is under investigation. Recent studies in FKRP mutant mice (Cataldi et al., 2018) and patient-specific iPS cell derivatives (Nickolls et al., 2020; Ortiz-Cordero et al., 2021b) show the partial restoration of α-DG functional glycosylation upon treatment. An open-label study was announced in March 2021 to determine the safety and tolerability of ascending dose levels of BBP-418 (ribitol) for the treatment of ambulatory and non-ambulatory LGMDR9 patients (NCT04800874). Although encouraging, this study is still in the very early stages.

iPS cells hold great potential for the development of cell-based treatments for several degenerative diseases. Clinical trials are being designed or are already under way with PS cell derivatives, including the transplantation of pigment epithelial sheet for the treatment of macular degeneration (Araki et al., 2019; Jha et al., 2021) (NCT04339764), dopaminergic neurons for Parkinson’s disease (Studer, 2017), and NK cells as an immunotherapy regimen for the treatment of solid tumors and hematological disorders (Cichocki et al., 2020; Hermanson et al., 2016) (NCT04106167, NCT04551885, NCT03841110, NCT04245722, and NCT04023071). All of these initiatives are encouraging, and as other investigators begin to develop and manufacture iPS cell-based therapeutic cell products, continuous effort must be made to make sure this is devoid of residual undifferentiated PS cells that can lead to undesired tumor formation. Another important point to consider when using gene editing is the risk of off-target mutagenesis. The assessment performed here showed a lack of CRISPR-Cas9-induced off-target mutations at selected sites in gene-corrected cells, but a more comprehensive and unbiased analysis will be required to establish the safety of this approach before this can be applied therapeutically. An advantage of our CRISPR platform is its clinical relevance as we also show that this approach can be adapted to a safer, scar-free, selection-cassette-free approach.

In summary, we have developed and validated a universal gene-editing platform for correcting FKRP-associated MDs in multiple patient-derived iPS cell lines. This CRISPR-Cas9 and HDR approach can also be applied to generate patient-specific mutations in iPS cells, extending its application for disease modeling. Before this gene-correction strategy can be used for the development of an autologous cell therapy, further studies are required to address aspects related to manufacturing, scalability, biodistribution, safety, efficacy, and delivery. These are critical prerequisites to move this therapeutic approach toward clinical translation.

## STAR★METHODS

### RESOURCE AVAILABILITY

#### Lead contact

Further information and requests for resources and reagents should be directed to and will be provided by the lead contact, *Rita C.R. Perlingeiro* (perli032@umn.edu).

#### Materials availability

Plasmids and cell lines generated in this study will be provided upon request to *Rita C.R. Perlingeiro* (perli032@umn.edu).

#### Data and code availability

- Data reported in this paper will be shared by the lead contact upon request.
- This paper does not report original code.
- Flow cytometry, confocal and inverted microscopy raw data files reported in this paper will be shared by the lead contact upon request. Any additional information required to reanalyze the data reported in this paper is available by the lead contact upon request.

### EXPERIMENTAL MODEL AND SUBJECT DETAILS

#### iPS cell culture

Studies involved fibroblast samples according to procedures approved by the Institutional Review Board of the Sanford Burnham Prebys Medical Discovery Institute. FP3 and FP4 fibroblasts were obtained from 5 and 1 year old male patient reprogrammed into iPS cells using Cytotune 2.0 Sendai virus based reprogramming kit (Thermo Fisher Scientific) (Kava et al., 2013). iPS cell clones exhibiting typical iPS cell morphology and expression of pluripotency markers were selected for further studies. CDI73 iPS cells were kindly provided by Cellular Dynamics, Inc. (Madison, WI). As controls, we used iPS cell lines generated from unaffected individuals and previously validated in our laboratory (Baghbaderani et al., 2015; Darabi et al., 2012; Selvaraj et al., 2019c). iPS cells were maintained on matrigel-coated plates in the presence of mTeSR1 medium (STEMCELL Technologies) and passaged with ReLeSR (STEMCELL Technologies) or Accutase (Innovative Cell Technologies).

#### Mice studies

Animal experiments were carried out according to protocols approved by the University of Minnesota Institutional Animal Care and Use Committee. For the teratoma assay, human iPS cells were injected into the quadriceps of 8-week-old male immunodeficient mice (NSG; Jackson). Prior to injection, 1.5 × 10^6^ cells were resuspended in 50 μL of 1:1 solution of DMEM/F12: Matrigel. Teratomas were collected at 2 months post-injection, fixed, sectioned, and processed for hematoxylin-eosin staining. As previously described (Azzag et al., 2020) The FKRP^P448L^ mouse model was procured from Jackson Laboratories. To create an immunodeficient FKRP^P448L^ mouse model, the FKRP mutant was crossed with NSG (NOD/SCID; IL2 receptor gamma). Our analysis as previously reported (Azzag et al., 2020) showed lack of all of lymphocytes in these mice.F1 males (carrying gamma-c, which is X-linked) were backcrossed, and PCR was utilized to identify N1 pups having FKRP mutations and homozygous for NOD/SCID and IL2Rg. For transplantation studies, one day before cell injection, tibialis anterior (TA) muscles of 4 to 6 weeks old (male or female) FKRP^P448L^-NSG mice were pre-injured with 15 μl of cardiotoxin 10 μM (Latoxan) as previously described (Azzag et al., 2020). Myogenic progenitors (described below) were harvested using TryplE (GIBCO), washed with PBS, and injected at a density of 1 × 10^6^ cells (resuspended in 15 μL of PBS) using a 22 g Hamilton syringe. As control, the contralateral leg was injected with 15 μL of PBS. PBS-injected contralateral TA muscles served as negative controls. Six weeks post-transplantation, mice were euthanized and TAs collected for immunostaining analysis.

### METHOD DETAILS

#### Cell culture and myogenic differentiation

For myogenic differentiation, iPS cells were transduced with pSAM2-iPAX7-ires-mCherry and FUGW-rtTA lentiviral vectors and differentiated into myotubes, as previously described (Darabi et al., 2012; Selvaraj et al., 2019c). Transduced iPAX7 iPS cells were passaged at 90% density into single cells using Accutase, and further plated at a density of 1 × 10^6^ cells onto 60 mm non-adherent Petri dishes in mTeSR1 with 10 μM ROCK inhibitor Y-27632, on a shaker at 60 RPM and incubated at 37°C, to generate embryoid bodies (EBs). After two days, mTeSR1 was replaced with EB myogenic medium, which consists of Iscove’s Modified Dulbecco’s Medium (IMDM) supplemented with 15% FBS, 10% horse serum, 1% KnockOut Serum Replacement (KOSR; GIBCO), 10 μM GSK3b inhibitor (CHIR 990217; Tocris), 50 μg/ml ascorbic acid, 4.5 mM monothioglycerol, 1% GlutaMax, and 1% penicillin-streptomycin (GIBCO). After 48 hr, the existing medium was changed with fresh EB myogenic medium supplemented with 200 nM LDN-193189 and 10 mM SB-431542 (both from Cayman Chemical), as recently described (Selvaraj et al., 2019c). To induce PAX7 expression, 1 μg/ml of doxycycline (dox; Sigma-Aldrich) was added to the existing medium. After 24 hr, the medium was replaced with fresh dox-containing EB myogenic medium, and incubated for additional 48 hr. A small portion (about 1/10th) of the EBs was then centrifuged and were cultured on gelatin-coated T75 flasks in EB myogenic medium supplemented with dox and 5 ng/ml bFGF (PeproTech). EBs were maintained as a monolayer for four days and then harvested using Trypsin-EDTA solution and FACS sorted for mCherry to purify PAX7 positive myogenic progenitor cells. Sorted mCherry positive myogenic progenitors were plated on gelatin-coated T75 flasks in EB myogenic medium supplemented with dox and 5 ng/ml bFGF (PeproTech), and expanded for 3–4 passages. For terminal differentiation, myogenic progenitors were cultured to 100% confluency in low nutrient differentiation medium consisting of KnockOutTM DMEM, containing 20% KOSR, 10 μM each of SB-431542, DAPT, Forskolin, and Dexamethasone (Cayman Chemical and Selleckchem), 1% non-essential amino acids, 1% glutamax, and 1% penicillin-streptomycin.

#### CRISPR-Cas9-mediated genome editing

The HDR donor vector was constructed in the pBluescript plasmid backbone, as previously described (Selvaraj et al., 2019a). The 5′ homology arm consisting of intron 3 (~1000 bp) and 3′ homology arm consisting of the exon 4 untranslated region (~1000 bp) were PCR amplified and cloned upstream and downstream of GFP-2A-neoR cassette, respectively. Next, we cloned exon 4 wild-type or mutant cDNA and a SV40 polyA signal sequence upstream of GFP-2A-neoR cassette and downstream of 5′ homology arm. For the scar-free editing approach, we PCR amplified the region encompassing 5′ homology arm, cDNA and 3′ homology arm from a wild-type iPS cell line and cloned it in pBluescript plasmid backbone without the selection cassette. Gene editing of all iPS cells was carried out using a ribonucleoprotein (RNP) based delivery of guide RNA and Hifi Cas9 protein obtained from Synthego and IDT respectively. Sequences of guide RNAs used are as follows: 5′ gRNA CCGCATGGGGCCGAAGTCTG and 3′gRNA ACCCCCGAAAAACAAAGGCG. 24 hr prior to transfection, iPS cells were passaged as single cells and seeded at 75 × 10^3^ cells per well on a 24-well plate. CRISPR-Cas9 RNP complex and the HDR donor vector were transfected using Lipofectamine CRISPRMAX Cas9 Transfection Reagent (Thermo Fisher Scientific) as per manufacturer’s instructions. 72 hr later, transfected cells were passaged onto a 6 well plate, and 48 hr later, the first round of antibiotic selection was started by adding Geneticin (G418) at 50 μg/ml (Thermo Fisher Scientific) for 10 days. Upon cell passaging, we initiated antibiotic selection for 5 days with Ganciclovir (4 μM; InvivoGen) to select against random integration of the donor vector. The iPS cell colonies that survived this double selection were then plated sparingly with cloneR (STEMCELLTechnologies) to obtain single-cell clones. We picked approximately 30 clones for each cell line. Genomic DNA PCR was performed with PrimeStar GXL DNA polymerase (Takara) using primers that bind to the genomic DNA upstream and downstream of the homology arms forward primer-GAATGTGGAGGGGAGTGTCCTAAGGTT and reverse primer CTGCTAAGTGGGTCTCCAAGCCCC. Clones that were positive for this PCR were selected for further analysis. Next we performed Knock in-specific PCR using forward primer GAATGTGGAGGGGAGTGTCCTAAGGTT and reverse primer TGGCGGCAAACCC GTTGCGAAAAAGA.

#### RT-PCR analysis

Cells were lysed using Trizol reagent (Thermo Fisher) and RNA was extracted using the PureLinkTM RNA mini kit (Thermo Fisher) with on-column DNase treatment following manufacturer’s instructions. RNA concentration was quantified using Nanodrop. SuperScript VILO cDNA synthesis kit (Thermo Fisher Scientific) was utilized to perform reverse transcription of the RNA. RT- PCR was performed using an amount of cDNA corresponding to 20–50 ng of starting RNA with the PrimeStar GXL DNA polymerase (Takara) as per manufacturer’s instructions. Following are the RT-PCR amplicons and their corresponding primer sequences, FKRP exon 4-Poly SV40 (FP:TGCCCGAGCTGGTAGACTCC RP: CACACCTCCCCCTGAACCTG), FKRP Exon 4(FP:TGCCCGAGCTGGTAGACTCC RP: CCCAGCTCACTAGGCGGATG), ACTB (FP:GCGACGAGGCCCAGAGCAAG RP: TGGCCGTCAGGCAGCTCGTA).

#### FACS for IIH6

iPS cells were dissociated using enzyme-free dissociation buffer, collected, centrifuged at 500 g, and resuspended in FACS buffer consisting of 10% FBS and 1% penicillin/streptomycin in PBS. Fc Block reagent was added at 1 μl/million cells (BD Bioscience), and incubated for 5 min. Following the blocking step, staining was performed either with 1 μl of normal mouse IgM antibody (Santa Cruz Biotechnology) or anti-α-DG antibody IIH6C4 (Millipore) in 200 μl of FACS buffer per million cells for 20 min on ice. After washing with PBS, cells were resuspended in 200 μl of FACS buffer and incubated with either Alexa flour 488- or 647-conjugated anti-mouse IgM secondary antibody (1:500) for 20 min on ice. Cells were washed, resuspended in FACS buffer, filtered through 35 μm FACS tubes, and analyzed using a FACS Aria II (BD Biosciences).

#### Western blot

Protein extraction and western blot analysis were performed as described previously (Azzag et al., 2020). Briefly, cells were washed with PBS, scraped, and total protein was extracted via homogenization in Tris-Buffer Saline (TBS, 50 mM Tris-Cl, pH 7.5, 150 mM NaCl) with 1% Triton X-100 supplemented with protease inhibitor cocktail (Complete – Millipore Sigma) at 4°C for 30 min. The supernatant was collected by centrifugation for 30 min at 14000 g and concentration was determined by Bradford assay (Millipore-Sigma). 50 μg of the total protein was then electrophoresed on a 4%–20% then transferred to a PVDF membrane. The PVDF membrane was blocked with 5% nonfat dry milk in PBS with Tween 20 (PBST) for 1 hr, and then were incubated with primary antibodies IIH6C4 (1:1000), β-DG (1:500) and MF-20 (1:100) overnight at 4°C. Anti-mouse IgG/IgM HRP conjugated secondary antibodies (Abcam) were applied at a dilution of (1:10000) for 1hr and the protein detection was performed using the Supersignal West Femto chemiluminescent substrate (Thermo Fisher Scientific) and was imaged in chemidoc imager (Bio-Rad).

#### WGA pull-down for glycosylated α-DG and laminin overlay assay

To perform WGA pull-down, 400 ug of protein lysate was used and incubated with 40 μl of Wheat Germ Agglutinin bound agarose beads (Vector Laboratories, Inc.) and incubated overnight at 4°C on a rotor. The protein beads conjugate was washed with TBS and 0.1% Triton X-100 and was eluted with 2x LSB and incubated at 100°C for 5 min. Protein samples were separated on 4%–20% SDS polyacrylamide gel and transferred to PVDF and were detected by ECL using a Bio-Rad imaging system as described above. The laminin-binding assay was performed as previously described, with minor modifications (Azzag et al., 2020). The WGA purified samples were electrophoresed using 4%–20% SDS-polyacrylamide gradient gels. Following transfer of proteins to PVDF membrane, this was blocked with 5% nonfat dry milk for 1 hr, briefly washed with TBS and then incubated with TBS, containing 1 mM CaCl2, 1 mM MgCl2 (TBSS), 3% BSA and 1 mg/ml native laminin (L2020 Sigma) for 2 hr at room temperature. Next, the membrane was washed twice for 10 min with TBSS and incubated overnight at 4°C with TBSS containing 3% BSA and anti-Laminin (L9393 Sigma). Afterward, the membrane was washed with TBSS twice for 10 min and incubated with HRP conjugated anti-rabbit IgG for 1hr. Finally, membranes were washed with TBSS twice for 10 min and the signal was detected using the chemiluminescence substrate as described above.

#### Off-target analysis

For this, we analyzed the top 5 predicted off-target regions for 5′ and 3′ guide RNA targeting exon 4, as predicted by the Off-Spotter software (Pliatsika and Rigoutsos, 2015). The genomic regions were PCR amplified in unedited and edited FP4, CDI73, FP3 iPS cells, and the amplicons were subjected to Sanger sequencing. Next, the chromatograms were compared using the ICE software to determine the percentage of off-target editing as per the manufacturer’s instructions (Hsiau et al., 2019). This software estimates INDEL frequencies upon CRISPR-Cas9 induced double-stranded break in a specific genomic site. Based on the incidences of modification in sequencing chromatograms around the CRISPR-Cas9 cut site, ICE software predicts the off-target frequency in the genome-edited sample using the unedited sample as the baseline control.

#### Karyotype analysis

Karyotype analysis was performed at the Cytogenomics lab at the University of Minnesota. iPS cells were harvested and chromosomes were analyzed by Giemsa banding at a resolution of 400–450 band level.

#### Immunostaining

Upon collection, muscles were frozen in isopentane cooled in liquid nitrogen, and serial 10–15 μm-thick cryosections were collected. Cells were washed with PBS and fixed with 4% PFA for 20 min at RT. Next, cells were permeabilized with 0.3% Triton X-100 in PBS for 20 min at RT followed by blocking with 3% BSA for 1 hr at RT and then incubated with primary antibody diluted in 3% BSA overnight at 4°C. Cells were then washed and incubated with secondary antibody diluted in blocking solution supplemented with DAPI for 1 hr at RT. Cells were washed with PBS and were maintained in PBS until the final analysis. Images were acquired using an inverted fluorescence microscope (Zeiss) or were analyzed by confocal microscopy (NikonNiE C2 upright confocal microscope). For muscle tissues, cryosections were fixed with 4% PFA for 10 min and permeabilized with 0.3% Triton X-100 in PBS for 20 min at RT, then blocked and incubated with primary antibodies overnight. The next day, slides were washed and incubated with the secondary antibodies, washed again, and were then mounted with coverslips using ProLong Gold Antifade Mountant with DAPI (Thermo Fisher Scientific). Following are the antibodies used for immunofluorescence staining: human-α-dystroglycan IIH6C4 (1Millipore; 1:200. and DSHB; 1:50), human DYSTROPHIN (MANDYS106, DSHB; 1:50), human LAMIN A/C (ab108595, Abcam; 1:500), MHC (MF20, DSHB; 1:100), OCT3/4 (C-10; 1:50), SOX2 (Y-17; 1:50), NANOG (H-2; 1:50), (all from Santa Cruz Biotechnology), Alexa Fluor 488 anti-rabbit IgG (A-11008; 1:500), Alexa Fluor 647 goat anti-mouse IgG (A-21235; 1:500) and Alexa Fluor 488 goat anti-mouse IgM A-10680; (1:500) and Alexa Fluor 555 goat anti-mouse IgG (A21424; 1:500) (all from Thermo Fisher Scientific).

### QUANTIFICATION AND STATISTICAL ANALYSIS

Details of experiments, including number of samples, can be found in the figure legends. Results represent the average of biological or technical replicates ± standard error. For comparisons of multiple groups, we used the one-way ANOVA followed by the Tukey’s test. *p* values < 0.05 were considered significant. Statistical analysis was performed using GraphPad Prism (GraphPad Software).

## Supplementary Material

1

## Figures and Tables

**KEY RESOURCES TABLE:** 

REAGENT or RESOURCE	SOURCE	IDENTIFIER
Antibodies		
Anti-alpha dystroglycan (mouse monoclonal)	Millipore	Cat# 05–593; RRID: AB_309828
Anti-alpha dystroglycan (mouse monoclonal)	DSHB	Cat# IIH6 C4; RRID: AB_261721
Anti-MHC (mouse monoclonal)	DSHB	Cat# MF20; RRID: AB_21477
Anti-laminin (rabbit polyclonal)	Sigma-Aldrich	Cat# L9393; RRID: AB_47716
Anti-beta dystroglycan, concentrated (mouse monoclonal)	DSHB	Cat# MANDAG2 clone 7D11; RRID: AB_221177
Anti-OCT3/4 (mouse monoclonal)	SCBT	Cat# C-10; RRID: AB_62805
Anti-SOX2 (goat polyclonal)	SCBT	Cat# Y-17; RRID: AB_228668
Anti-NANOG (mouse monoclonal)	SCBT	Cat# H-2; RRID: AB_1091825
Anti-SSEA4 (mouse monoclonal)	SCBT	Cat# sc-21704; RRID: AB_62828
Alexa fluor 555 goat anti-mouse IgG (goat polyclonal)	Thermo Fisher Scientific	Cat# A-21424; RRID: AB_141780
Alexa fluor 647 goat anti-mouse IgM (goat polyclonal)	Thermo Fisher Scientific	Cat# A-21238; RRID: AB_2535807
Goat Anti-Mouse IgG+IgM H&L (HRP) preadsorbed	Abcam	Cat# ab47827; RRID: AB_955398
Bacterial and virus strains		
One shot Top 10	Thermo Fisher Scientific	Cat# C404006
Chemicals, peptides, and recombinant proteins		
CHIR99021	Tocris	Cat# 4423
LDN193189	Cayman chemical	Cat# 19396
SB431542	Cayman chemical	Cat# 13031
DAPT	Cayman chemical	Cat# 13197
Dexamethasone	Cayman chemical	Cat# 11015
Forskolin	Cayman chemical	Cat# 11018
Doxycycline	Sigma-Aldrich	Cat# D989
Geneticin Selective Antibiotic (G418 Sulfate)	Thermo Fisher Scientific	Cat#10131035
Ganciclovir (GCV)	InvivoGen	Sud-gcv
Recombinant human FGF-basic	Peprotech	Cat# 100–18
Experimental models: cell lines		
FP4	This study. Available upon request: Anne Bang, Sanford Burnham Prebys Medical Discovery Institute	FP4
FP3	This study. Available upon request: Anne Bang, Sanford Burnham Prebys Medical Discovery Institute	FP3
CDI73	FUJIFILM Cellular Dynamics, Inc.	CDI73
Control 1	PMID:22560081 available from the Rita Perlingeiro lab	PLZ
Control 2	PMID:26411904 available from the RUCDR Infinite Biologics	TC1133
Control 1 MUT C7	This study. Available upon request: Rita Perlingeiro, University of Minnesota	PLZ-C7
Control 1 MUT C12	This study. Available upon request: Rita Perlingeiro, University of Minnesota	PLZ-C12
Control 2 MUT C8	This study. Available upon request: Rita Perlingeiro, University of Minnesota	TC1133-C8
Control 2 MUT C12	This study. Available upon request: Rita Perlingeiro, University of Minnesota	TC1133-C12
FP4 C40	This study. Available upon request: Rita Perlingeiro, University of Minnesota	FP4-corrected
FP4 Scar-Free	This study. Available upon request: Rita Perlingeiro, University of Minnesota	FP4-SF-corrected
CDI73 C22	This study. Available upon request: Rita Perlingeiro, University of Minnesota	CDI73 HDR C22
FP3 C6	This study. Available upon request: Rita Perlingeiro, University of Minnesota	FP3 HDR C6
Experimental models: organisms/strains		
NSG	Jackson Laboratories	NSG™ (NOD.*Cg-Prkdc*^*scid*^*Il2rg*^*tm1Wjl*^/SzJ (005557)
FKRP-NSG	Azzag et al., 2020	FKRP^P448L^-NSG
Oligonucleotides		
5′ gRNA CCGCATGGGGCCGAAGTCTG	Synthego	N/A
3′gRNA ACCCCCGAAAAACAAAGGCG	Synthego	N/A
Full length FP: GAATGTGGAGGGGAGTGTCCTAAGGTT	This study	N/A
Full Length RP: CTGCTAAGTGGGTCTCCAAGCCCC	This study	N/A
Knock in-specific PCR FP: GAATGTGGAGGGGAGTGTCCTAAGGTT	This study	N/A
Knock in-specific PCR RP: TGGCGGCAAACCC GTTGCGAAAAAGA	This study	N/A
FKRP exon 4-Poly SV40 FP:TGCCCGAGCTGGTAGACTCC	This study	N/A
FKRP exon 4-Poly SV40 RP: CACACCTCCCCCTGAACCTG	This study	N/A
FKRP Exon 4 FP:TGCCCGAGCTGGTAGACTCC	This study	N/A
FKRP Exon 4 RP: CCCAGCTCACTAGGCGGATG	This study	N/A
ACTB FP:GCGACGAGGCCCAGAGCAAG	This study	N/A
ACTB RP: TGGCCGTCAGGCAGCTCGTA	This study	N/A
Recombinant DNA		
HDR Donor Vector	This study	Intron 3 5′HA-Exon 4 FKRP Exon4 3′HA
HDR Mutant Donor Vector	This study	Intron 3 5′HA-Exon 4-FKRP SF Exon4 3′HA
HDR Scar Free Donor Vector	This study	Intron 3 5′HA-Exon 4-FKRP MUT Exon4 3′HA
Software and algorithms		
ImageJ	Schneider et al., 2012	https://imagej.nih.gov/
Zen Lite	Zeiss Microscopy	https://www.zeiss.com/microscopy/us/products/microscope-software/zen-lite.html
GraphPad Prism	GraphPad Software, LLC	RRID: SCR_002798 https://www.graphpad.com
TIDE: Tracking of Indels by DEcomposition	Brinkman et al., 2014	https://tide.nki.nl/

## Highlights

- Defective α-DG functional glycosylation in patient-specific FKRP mutant myotubes
- Universal CRISPR-Cas9-mediated gene-editing approach to correct FKRP mutations
- Rescue of α-DG functional glycosylation in gene-corrected FKRP myotubes
- Donor-derived myofibers show rescue of α-DG functional glycosylation in FKRP mice
