# Supplementary material for: A universal gene correction approach for FKRP-associated dystroglycanopathies to enable autologous cell therapy
Source: Cell Rep. Author manuscript; Available in PMC 2021 Aug 2. (PMC8327854; doi:10.1016/j.celrep.2021.109360)
Supplement: 1 [file NIHMS1724256-supplement-1.pdf]

Cell Reports, Volume *36*

Supplemental information

A universal gene correction approach for FKRP-associated dystroglycanopathies to enable autologous cell therapy

Neha R. Dhoke, Hyunkee Kim, Sridhar Selvaraj, Karim Azzag, Haowen Zhou, Nelio A.J. Oliveira, Sudheer Tungtur, Carolina Ortiz-Cordero, James Kiley, Qi Long Lu, Anne G. Bang, and Rita C.R. Perlingeiro

Figure S1 related to 1

A

iPS cell line (Phenotype)	Age	Nucleotide change	Protein change
FP4 (WWS)	<1year	c.558dupC<br>c.1418T>G	p.Ala187Fs<br>p.Phe473Cys
CDI73 (LGMD2R9)	Adult	c.826C>A<br>c.826C>A	p.Leu276Ile
FP3 (LGMD2R9/CMD)	15 months	c.217C>T<br>c.826C>A	p.Glu73 OCH<br>p.Leu276Ile

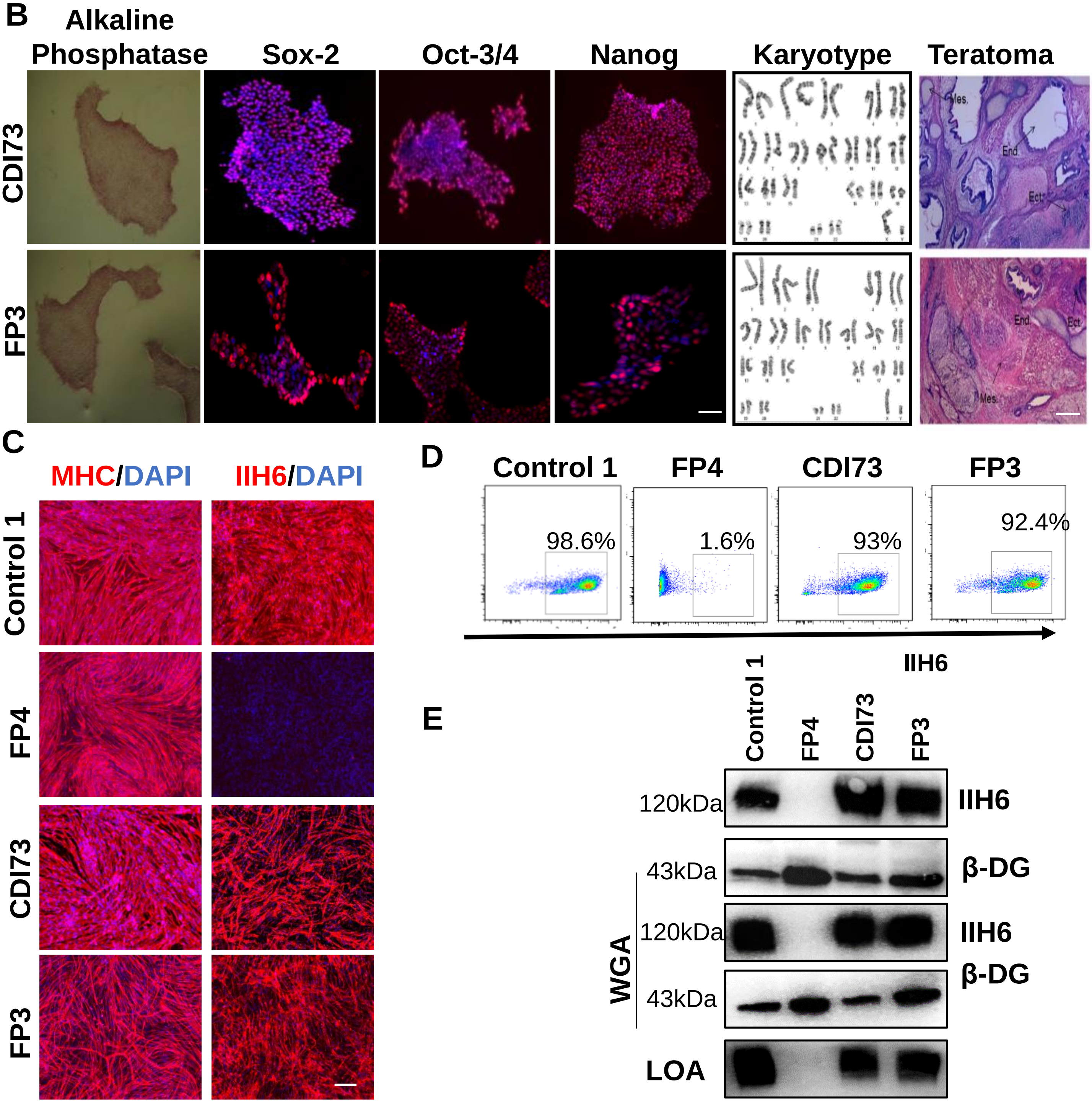

Figure S1 related to 1. Characterization of FKRP mutant patient-specific iPS cells and respective myotubes.

(A) Information of FKRP mutant patient-specific iPS cell lines.

(B) Pluripotency characterization of CDI73 and FP3 iPS cell lines. Representative images show alkaline phosphatase activity and immunostaining for OCT3/4, SOX2, and NANOG (red). DAPI stains nuclei (in blue). Scale bar, 200 μm. Cytogenetic analyses show normal karyotype. Representative images show hematoxylin-eosin staining of teratomas with contribution to all three germ layers, resulting from the intramuscular injection of CDI73, and FP3 iPS cells into NSG mice.

(C) Representative images show immunostaining for MHC (left) and IIH6 (right) in control and FKRP mutant iPS cell-derived myotubes. DAPI stains nuclei (in blue). Scale bar, 200 μm.

(D) Flow cytometry analysis for IIH6 in control and FKRP mutant patient-specific iPS cells.

(E) Representative western blot for IIH6 in in control and FKRP mutant patient-specific iPS cells. β-DG was used as loading control (upper panel). Middle panel shows WGA pull-down for these samples. Lower panel shows representative LOA of WGA elutes.

Figure S2 related to 1

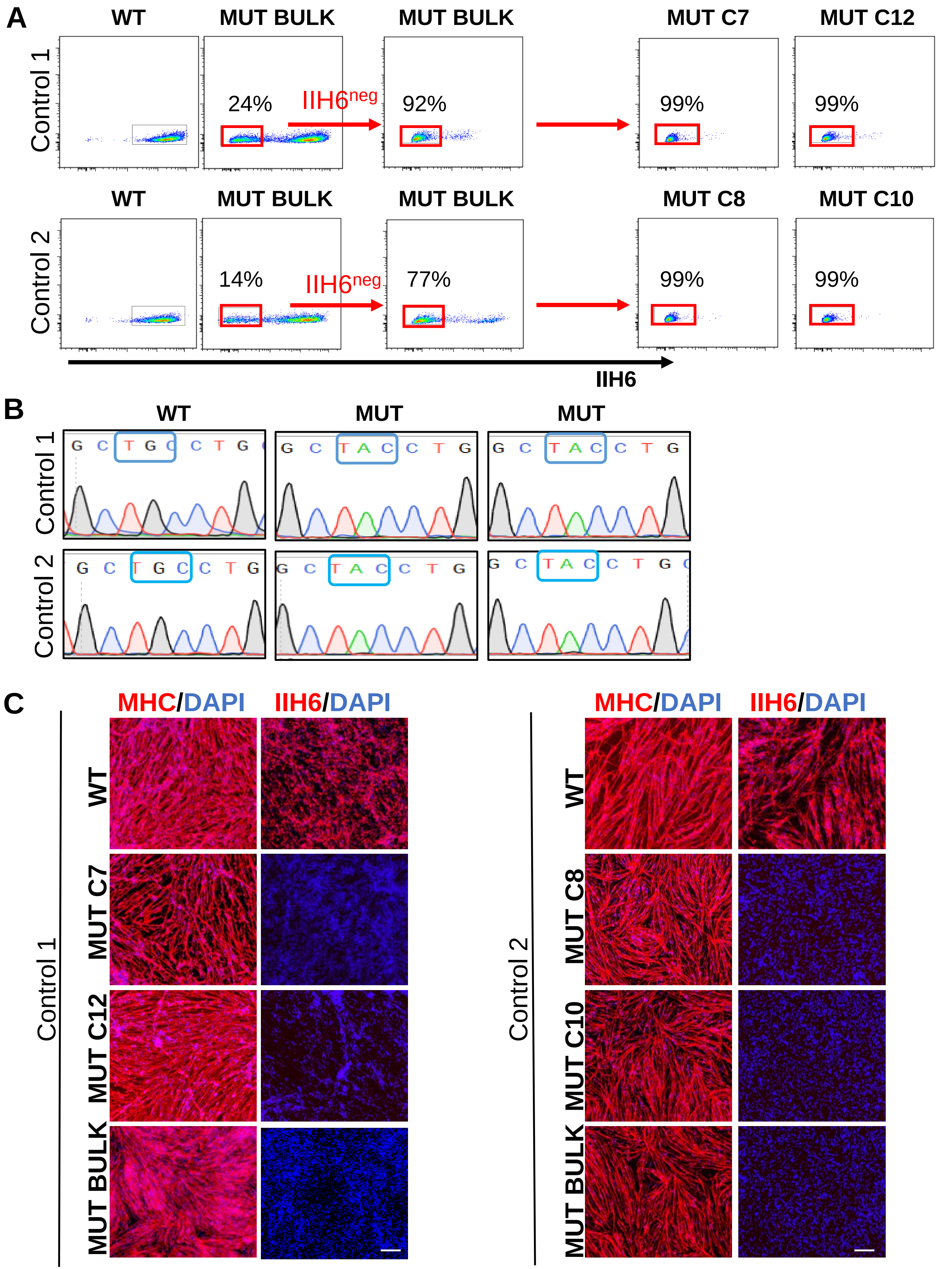

Figure S2 related to 1. Characterization of WWS-associated FKRP mutant isogenic iPS cell lines.

(A) Flow cytometry analysis for IIH6 in FKRP mutant isogenic iPS cells. Plots show results from control 1 (WT, mutant bulk, and clones C7 and C12) and control 2 (WT, mutant bulk, and clones C8 and C10) iPS cells. Following antibiotic selection, mutant bulk iPS cells from control 1 and control 2 were sorted based on the lack of IIH6 reactivity (18.5% and 14%, respectively). Following this enrichment, the vast majority of the bulk population for controls 1 and 2 was negative for IIH6 (90% and 77%, respectively). Following single cell cloning, two clones for each cell line were further analyzed: C7 and C12 for control 1, and C8 and C10 for control 2. These clones were found to be 99% negative for IIH6 (far right), thus confirming loss of functional α-DG glycosylation.

(B) Sequencing analysis confirmed mutation in WWS-associated FKRP mutant isogenic iPS cells. (C) Representative images show immunostaining for MHC (left) and IIH6 (right) in myotubes derived from control 1 (WT, mutant bulk, and clones C7 and C12) and control 2 (WT, mutant bulk, and clones C8 and C10) iPS cells. DAPI stains nuclei (in blue). Scale bar, 200 μm.

Figure S3 related to 3

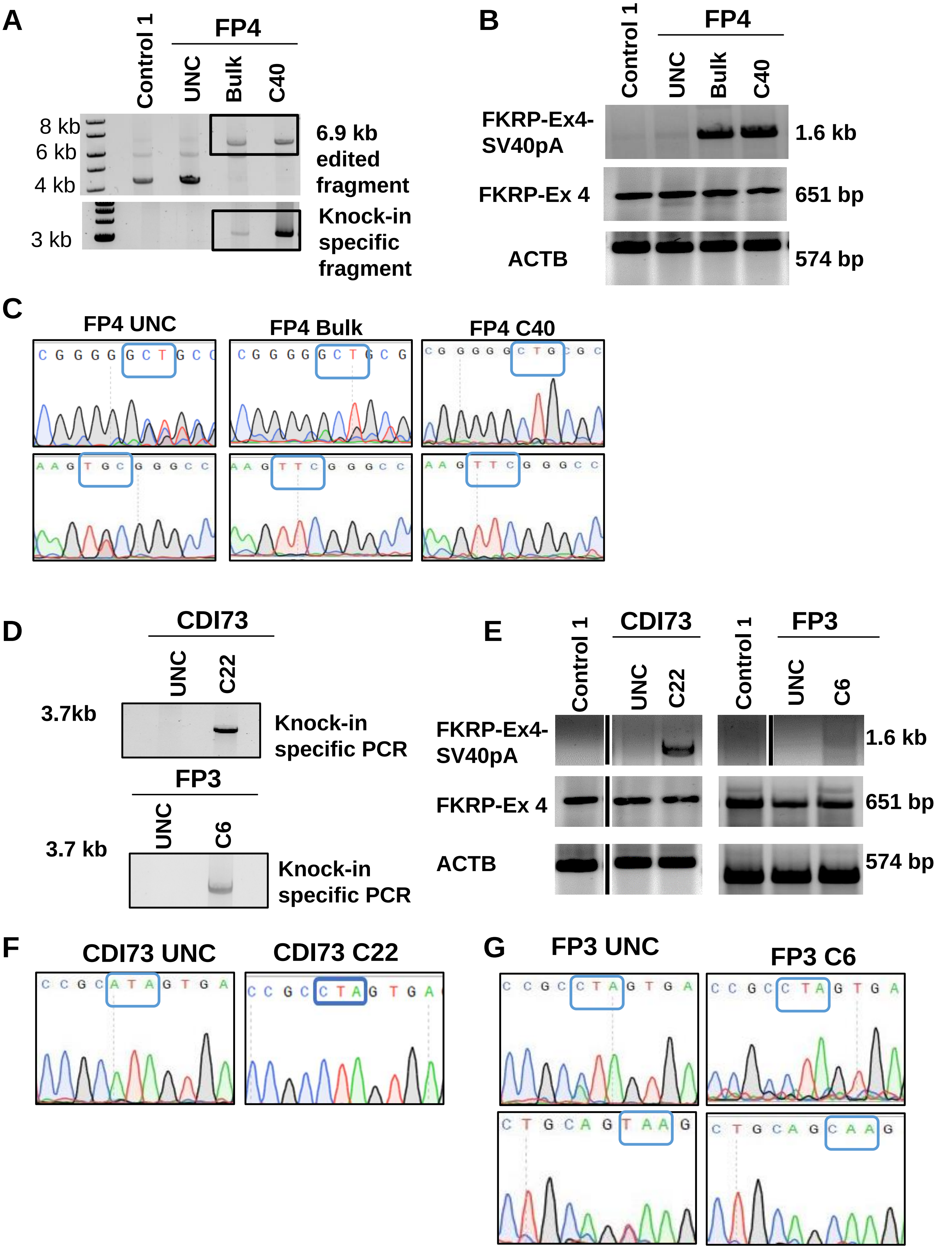

Figure S3 related to 3. Gene correction of FKRP mutations in FP4, CDI73 and FP3 iPS cells.

(A) Upper panel shows PCR amplification of the region encompassing the knock-in in genomic DNA, indicating that the knock-in is homozygous in both gene corrected bulk and clone 40 (C40) as indicated by the presence of a single 6.9kb edited amplicon. The presence of a second PCR amplicon of 4.2 kb would indicate heterozygosity. Uncorrected cells show the unedited PCR amplicon of 4.2 kb. Lower panel shows PCR amplification of the knock-in specific region in genomic DNA from uncorrected (UNC) and gene corrected FP4 (bulk and C40) iPS cells.

(B) RT-PCR analysis of gene corrected (bulk and C40) and uncorrected (UNC) FP4 iPS cells. Unaffected iPS cells served as controls. Upper panel shows amplification of FKRP exon 4 to SV40pA sequence, which is specific to the insert. The middle panel shows amplification of FKRP exon 4, which is present in all samples. The lower panel indicates ACTB used as loading control. (C) Sequencing chromatograms show correction of FKRP mutations in gene edited FP4 iPS cells. (D) PCR shows amplification of the knock-in specific region in genomic DNA from uncorrected (UNC) and gene corrected CDI73 (C22) and FP3 (C6) iPS cells.

(E) RT-PCR analysis of gene corrected CDI73 (C22) and FP3 (C6) iPS cells, uncorrected counterparts, and control iPS cells. Upper panel shows amplification of *FKRP* Exon 4 to SV40pA sequence, which is specific to the insert. The middle panel shows amplification of FKRP exon 4, which is present in all samples. The lower panel indicates *ACTB* used as loading control. The line indicates that these samples were not run on consecutive lanes in the gel.(F-G) Sequencing chromatograms show correction of FKRP mutations in gene edited CDI73 (F) and FP3 (G) iPS cells.

Figure S4 related to 3

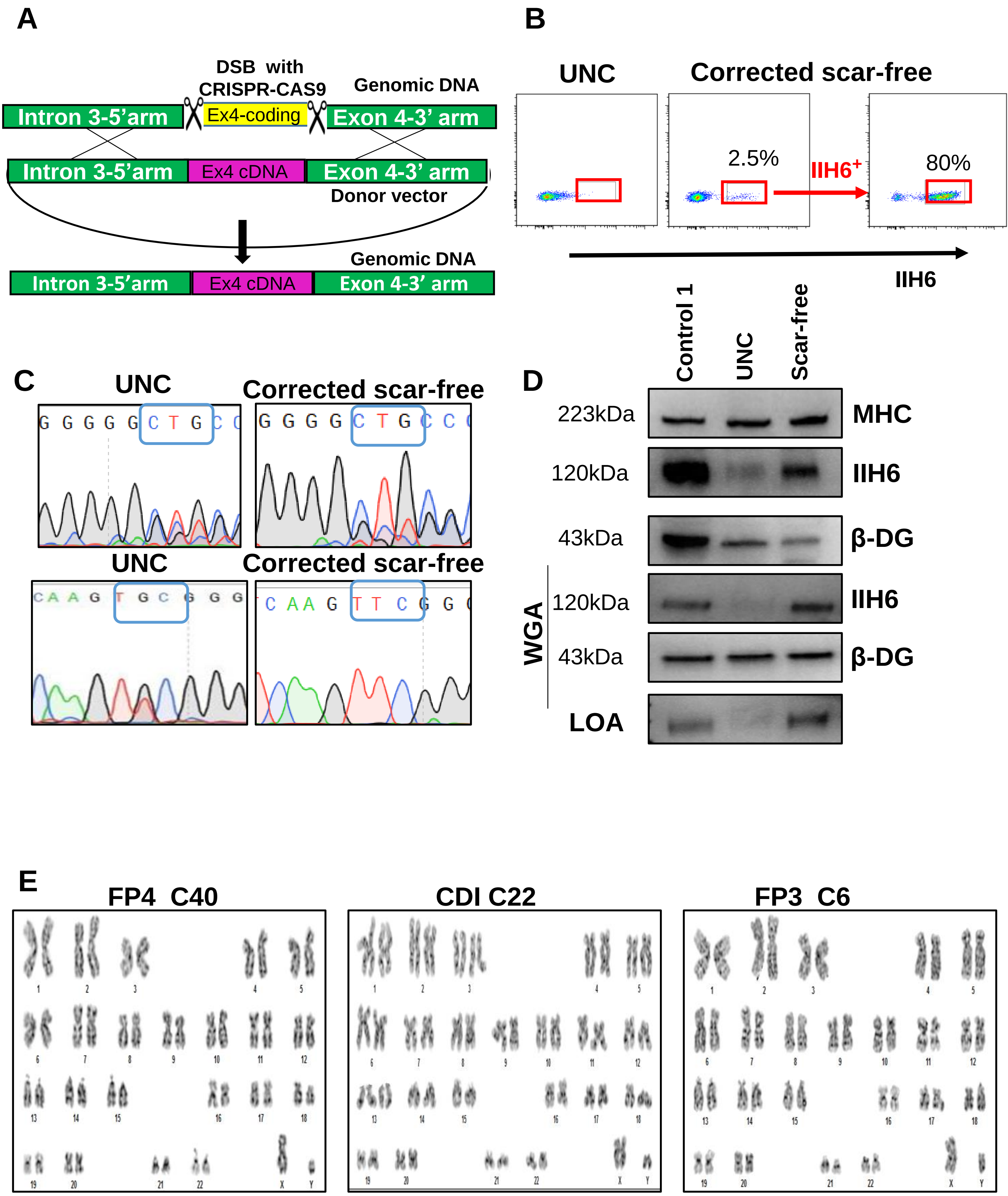

Figure S4 related to 3. Gene correction of FP4 iPS cells using a scar-free approach and Karyotype analysis

(A) Schematic of the scar-free gene correction approach for gene knock-in.

(B) Flow cytometry analysis for IIH6 in uncorrected (UNC) and gene edited FP4 iPS cells using the scar-free approach.

(C) Sequencing analysis confirmed the correction of scar-free gene edited FP4 patient-specific iPS cells.

(D) Western blot and LOA show rescue of functional α-DG glycosylation in scar-free gene corrected FP4 iPS cell-derived myotubes, as shown by IIH6 positivity and laminin binding (lower panel). MHC and β-DG were used as differentiation and loading controls, respectively.

(E) Karyotype analysis of gene corrected FP4 (C40), CDI73 (C22), and FP3 (C6) iPS cells.

Table S1 related to Figure 3. Off-target analysis in selected sites.

gRNA-PAM sequence 5'gRNA-FKRP	Gene (chr)	Predicted off-target percentage based on ICE analysis			
		FP4 bulk	FP4 C40	CDI73 C22	FP3 C6
CCGCATGGGGCtGctGTCTG	KCNK9 (Chr 9)	0%	0%	0%	0%
CCaCATGGGGCgGAtGTCTG	N/A (Chr 6)	0%	0%	0%	0%
CtGgATGGGGgCGgAGTCTG	ZNF784 (Chr 19)	0%	0%	0%	0%
CaGCcTGtGGCCGgAGTCTG	TBC1D9B (Chr 5)	0%	0%	0%	0%
aCGCATGGaGaaGAAGTCTG	N/A 1 (Chr 11)	0%	0%	0%	0%
**gRNA-PAM sequence 3'gRNA-FKRP**	**Gene (chr)**	**FP4 bulk**	**FP4 C40**	**CDI73 C22**	**FP3 C6**
tCCtgaGAAAAACAAAGGC	UE4B (Chr 1)	0%	0%	0%	0%
gCCtCCGAgAAACAgAGGCG	DOC2A (Chr 16)	0%	0%	0%	0%
gCCtCCGAAAAtCAtAGGCG	N/A (Chr 3)	0%	0%	0%	0%
cCCCCtGgAAgACAAAGGCG	TMSB15A (Chr X)	0%	0%	0%	0%
cCaCCCaAAAAAaAAAGGCG	N/A (Chr 16)	0%	0%	0%	0%

Table S1 related to 3. Off-target analysis in selected sites.

Analysis of sequencing chromatograms using the ICE tool showed no measurable off-target activity at selected sites (5 sites each for the two gRNAs) of gene corrected FP4 (bulk and C40), CDI73 (C22), FP3 (C6) iPS cell lines. Tables show the off-target analysis on top 5 predicted sites each for 5'gRNA and 3' gRNA. The off-target gRNA sequence, chromosome location (in brackets), and the gene names are listed.
